# Resolving the phylogenetic origin of glioblastoma via multifocal genomic analysis of pre-treatment and treatment-resistant autopsy specimens

**DOI:** 10.1038/s41698-017-0035-9

**Published:** 2017-09-18

**Authors:** Priscilla K. Brastianos, Naema Nayyar, Daniel Rosebrock, Ignaty Leshchiner, Corey M. Gill, Dimitri Livitz, Mia S. Bertalan, Megan D’Andrea, Kaitlin Hoang, Elisa Aquilanti, Ugonma N. Chukwueke, Andrew Kaneb, Andrew Chi, Scott Plotkin, Elizabeth R. Gerstner, Mathew P. Frosch, Mario L. Suva, Daniel P. Cahill, Gad Getz, Tracy T. Batchelor

**Affiliations:** 1 Division of Hematology/Oncology, Massachusetts General Hospital, Boston, Massachusetts USA; 2 Broad Institute of MIT and Harvard, Boston, Massachusetts USA; 3 Harvard Medical School, Boston, Massachusetts USA; 4 Division of Neuro-Oncology, Massachusetts General Hospital, Boston, Massachusetts USA; 5 Cancer Center, Massachusetts General Hospital, Boston, Massachusetts USA; 6 Laura and Isaac Perlmutter Cancer Center, NYU Langone Medical Center, New York, NY USA; 7 Department of Pathology, Massachusetts General Hospital, Boston, Massachusetts USA; 8 Department of Neurosurgery, Massachusetts General Hospital, Boston, Massachusetts USA

## Abstract

Glioblastomas are malignant neoplasms composed of diverse cell populations. This intratumoral diversity has an underlying architecture, with a hierarchical relationship through clonal evolution from a common ancestor. Therapies are limited by emergence of resistant subclones from this phylogenetic reservoir. To characterize this clonal ancestral origin of recurrent tumors, we determined phylogenetic relationships using whole exome sequencing of pre-treatment IDH1/2 wild-type glioblastoma specimens, matched to post-treatment autopsy samples (*n* = 9) and metastatic extracranial post-treatment autopsy samples (*n* = 3). We identified “truncal” genetic events common to the evolutionary ancestry of the initial specimen and later recurrences, thereby inferring the identity of the precursor cell population. Mutations were identified in a subset of cases in known glioblastoma genes such as *NF1*(*n* = 3), *TP53*(*n* = 4) and *EGFR*(*n* = 5). However, by phylogenetic analysis, there were no protein-coding mutations as recurrent truncal events across the majority of cases. In contrast, whole copy-loss of chromosome 10 (12 of 12 cases), copy-loss of chromosome 9p21 (11 of 12 cases) and copy-gain in chromosome 7 (10 of 12 cases) were identified as shared events in the majority of cases. Strikingly, mutations in the *TERT* promoter were also identified as shared events in all evaluated pairs (9 of 9). Thus, we define four truncal non-coding genomic alterations that represent early genomic events in gliomagenesis, that identify the persistent cellular reservoir from which glioblastoma recurrences emerge. Therapies to target these key early genomic events are needed. These findings offer an evolutionary explanation for why precision therapies that target protein-coding mutations lack efficacy in GBM.

## Brain cancer: Non-coding genomic changes fuel glioblastoma growth

Non-coding and structural alterations may be early drivers of brain cancer development. A team led by Priscilla Brastianos and Tracy Batchelor from Massachusetts General Hospital, Boston, USA, analyzed the genetic landscape of glioblastoma by comparing pre-treatment and autopsy tumor specimens from 12 patients who died of the aggressive brain cancer. They identified a common set of four genetic events that occurred early in the evolution of nearly every patient’s cancer: three losses or gains of chromosome regions or entire chromosomes, and mutations in the gene-activating promoter of TERT, which encodes an enzyme implicated in the cancer’s growth. The findings help explain why therapies that target protein-coding mutations don’t work in brain cancer when they do in other tumor types. They also point to new drug targets.

## Introduction

Glioblastoma (GBM) is the most common primary malignant brain tumor, with a poor prognosis. Therapies (including therapies that target specific alterations) that have shown efficacy in other cancers have failed in GBM. In the past 3 decades, only a single cytotoxic chemotherapeutic agent, temozolomide (TMZ), has been approved and widely used for GBM and this drug only modestly extends survival. Although the genomics of GBM at diagnosis have been extensively characterized^1–3^, the existence and identity of genomic drivers leading to GBM progression and recurrence remain elusive.

Starting from a normal cell, cancers evolve via multiple rounds of mutation, selection, and expansion.^4, 5^ Continued elaboration of this phylogenetic process within the growing cancer-cell population results in branched genetic variegation,^6^ whereby multiple cancer subclones relate to each other in a phylogenetic tree-like fashion.^7^ Consequently, cancer biospecimens are substantially heterogeneous both across different anatomical regions^8–11^ and within single cancer biopsies.^11–15^

GBM, when compared to many other cancers^16^, is a genetically heterogeneous disease. Multiregional sampling of GBM at a single timepoint commonly demonstrates significant intratumoral heterogeneity.^17–19^ Studies of matched pre-treatment and recurrent GBM after failure of therapy remain limited^20–22^ especially at the extremes of disease, in large part due to the logistical challenges associated with obtaining tissue at recurrence or the time of death. The ongoing evolutionary processes leading to GBM recurrence, and ultimately death of the patient, remain largely uncharacterized.

Our objectives were to comprehensively characterize intratumoral heterogeneity and evolutionary patterns in GBM over the entire course of clinical care, from initial diagnosis to time of death. We initiated a GBM autopsy program at Massachusetts General Hospital, which offers us the ability to compare the evolution of genetic changes at diagnosis, during treatment, and at the time of tumor progression and death. This served as the basis for a phylogenetic analysis of GBM throughout the disease course, as described herein.

## Results

We identified GBM patients from our autopsy tissue bank and acquired pre-treatment tissue from diagnosis and matched post-treatment autopsy tissue. We performed whole exome sequencing of 12 GBM cases for which we had tumor tissue separated by time (*n* = 10) and space (*n* = 2). Clinical characteristics of the 12-patient case series are shown in Table 1. We also used a PCR-based assay followed by next generation sequencing to evaluate the presence and cancer cell fraction of the *TERT* promoter mutation.

**Table 1:** Clinical characteristics of the 12-patient case series

Variable	Number of Patients
Mean Age (yrs)	62.8 ± 7.4
Mean Progression-Free Survival (yrs)	0.9 ± 0.8
Mean Overall Survival (yrs)	1.4 ± 1.0
Female: Male	4:8
**Presenting Symptoms**	
Headache	6 (50)
Nausea	1 (8)
Memory Loss	3 (25)
Weakness	5 (42)
Visual Deficit	2 (17)
Vomiting	1 (8)
Seizure	3 (25)
Systemic Metastases	2 (17)
**Location**	
Left: Right	3: 9
Frontal	4 (33)
Temporal	6 (50)
Parietal	2 (17)
**Surgery**	
Initial Surgery	12 (100)
Second Surgery	4 (33)
Third Surgery	2 (17)
Subtotal Resection	5 (42)
Gross Total Resection	6 (50)
Biopsy	1 (8)
**SNaPshot genotyping**	
Wildtype	11 (92)
TP53, 742 C > T (Arg248Trp)	1 (8)
MGMT methylated: unmethylated	4: 7
**Therapy**	
Radiotherapy	12 (100)
Concurrent temozolomide	11 (92)
Adjuvant temozolomide	12 (100)
Mean number of adjuvant temozolomide cycles	6.3 ± 4.0
Surgery at Progression	2 (17)
Radiotherapy at Progression	
Bevacizumab	11 (92)
CCNU Salvage Therapy	4 (33)
**Types of Inhibitors received**	
EGFR	4 (33)
HDAC	1 (8)
MTOR	2 (17)
MET	1 (8)
CXCR4	1 (8)
VEGF	1 (8)

Values are presented as the number of patients (%) unless indicated otherwise. Percentages represent the percentage within a row.

We sequenced to high depth (mean target coverage 81X for the exome, 21,459X for the *TERT* promoter Fluidigm assay). The mean non-synonymous mutation rate in the post-treatment autopsy samples (*n* = 12) was 1.78 mutations/Mb (range 1.04 to 2.63) and the mean non-synonymous rate in the pre-treatment samples (*n* = 9) was 1.19 mutations/Mb (range 0.81 to 1.50), consistent with prior reported mutation rates in GBM.^2^ There were no samples that detectably exhibited a ‘hypermutator phenotype’, as has been reported in a subset of GBMs^23^.

**Fig. 1:**
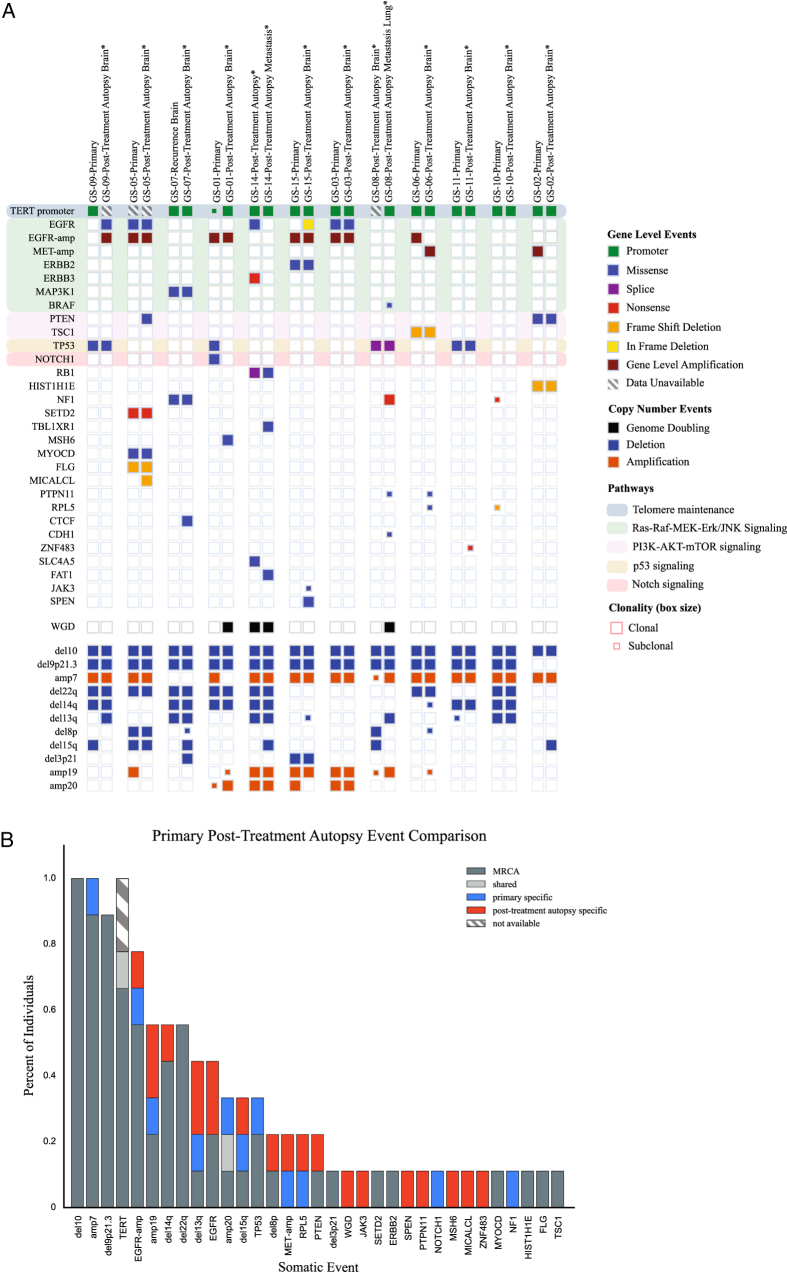
**a**. Comut plot of cohort. Columns are grouped together by individual (*n* = 12) in pairs. Both SNVs/indels (top panel) and copy number events (bottom panels) are included. Clonal and subclonal events are demarcated through the size of the box, with empty boxes specifying lack of presence of a mutation in that sample. Genes are grouped together by pathways with high relevance to glioblastoma found on the cBioPortal webpage (http://www.cbioportal.org/). **b**. Sample-specific bar plot. Only samples with both a pre-treatment primary and post-treatment autopsy sample were included (*n* = 9). Genetic aberrations (SNVs/indels and SCNAs) are represented in each bar, plotted categorically using categories MRCA (Most Recent Common Ancestor – clonal in both samples), shared (present in both samples, at subclonal levels in at least one sample), primary specific (present in primary sample, not present in post-treatment autopsy sample), post-treatment autopsy specific (present in post-treatment autopsy sample, not present in primary sample), not available (data not available – only applies to *TERT* promoter mutation where Fluidigm assay failed or was unavailable)

The most frequent point mutations were *TERT* promoter mutations, present in all patient cases where a Fluidigm assay was available (*n* = 11/11). For one case (GS-05), targeted sequencing data for the *TERT* promoter region was unavailable. Additionally, mutations that were previously reported in GBM were detected at a lower frequency compared to TERT alterations across our cohort, including mutations in *NF1* (*n* = 3 patients, 25% cases), *EGFR* (*n* = 5, 41.7% cases), *TP53* (*n* = 4, 33.3%), *RB1* (*n* = 1, 8.3%), *TSC1* (*n* = 1, 8.3%). The most frequent copy number alterations were loss of chromosome 10 and 9p21.3, as well as broad chromosome 7 gain, distinct from focal *EGFR* amplification (Fig. 1a).

We applied previously described computational methods^12, 24–27^ to address tumor heterogeneity and infer the evolutionary relationship between the matched, sequenced tissue samples from each patient. For each matched pre-treatment and post-treatment autopsy sample, we integrated copy-number alterations and somatic point mutation data to estimate a cancer-cell fraction (CCF) for each mutation, which were then analyzed to construct phylogenetic trees for clonality analysis to relate the cancer subclones within each patient (Fig. 1a, Supplementary Figure 1A-I).

All paired cases (temporally distinct pre- and post-treatment autopsy samples and spatially distinct metastatic autopsy samples) demonstrated a branched evolution pattern, whereby we detected a common ancestor (harboring truncal alterations), with each sample demonstrating significant subsequent genetic divergence. We noted a striking difference in the truncal status between coding alterations compared to non-coding and structural alterations. Phylogenetic reconstruction demonstrated that somatic exonic mutations, typically in the coding regions of common GBM driver genes, occurred on all branches of the phylogenetic tree, including in isolated subclonal branches (Fig. 1a). However, there were no protein-coding mutations identified as recurrent truncal events across the majority of the cohort. In contrast, despite variable clinical presentations and treatment courses, characteristic recurrent copy-number alterations and the *TERT* promoter mutation events were near-universally present clonally in the pre- and post-treatment autopsy samples (Fig. 1b). Chromosome 10 deletion was clonal in both matched samples in 9 of 9 cases (100%). Chromosome 7 copy-gain was clonal in the pre- and post-treatment autopsy samples in 8 of 9 cases (89%), vs. clonal uniquely in the pre-treatment sample in 1 case (11%). Chromosome 9p21.3 deletion was clonal in both matched samples in 8 of 9 cases (89%). *TERT* promoter mutations were clonal in both pre-treatment and post-treatment autopsy samples in 6 of 7 cases (86%) and subclonal in the pre-treatment and clonal in the post-treatment sample in 1 case (14%). In this latter case, the sample had clonal chromosome 10 and 9p21.3 loss in the pre-treatment and post-treatment autopsy samples, implying that the *TERT* promoter mutation can occur during gliomagenesis, after chromosome gains and losses. We were unable to infer clonality of *TERT* promoter mutations in the remaining 2 cases due to whole exome sequencing data deriving from a different biopsy sample than the sample used for the Fluidigm assay.

**Fig. 2:**
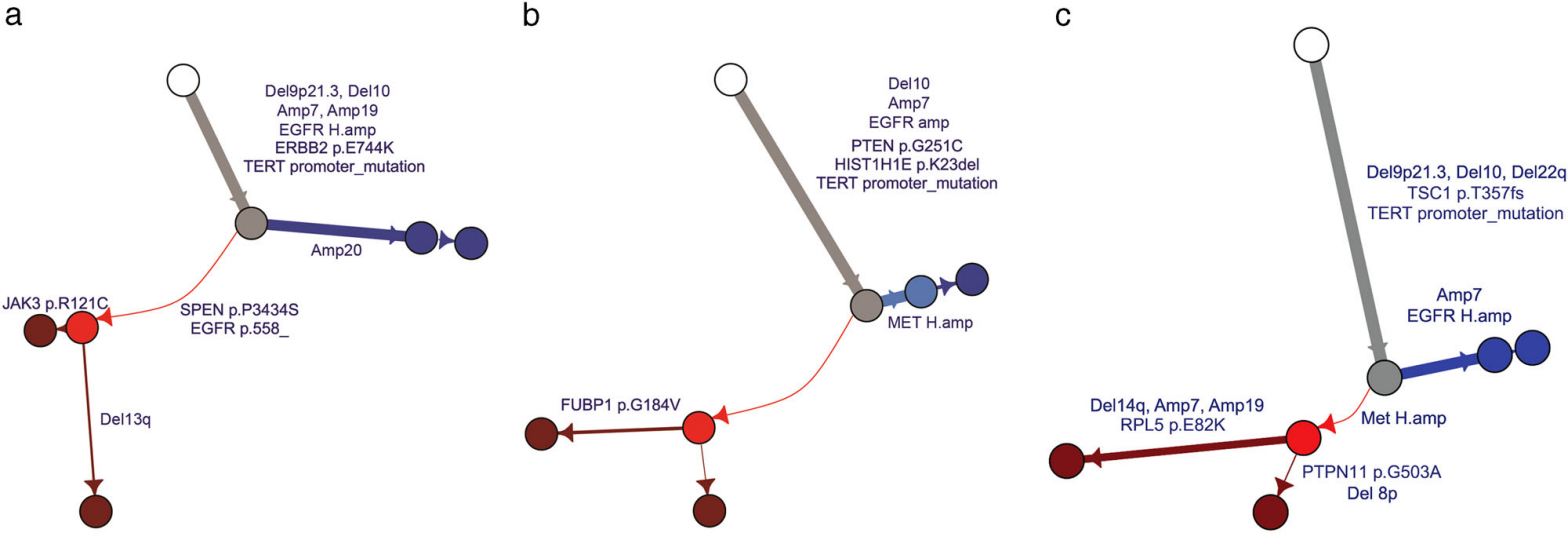
**a-c**. Phylogenetic Trees. Phylogenetic trees from representative cases with primary and post-treatment autopsy sample from the same individual. Primary specific clones occur on blue branches, and post-treatment autopsy specific clones occur on red branches, with mutations on driver genes and SCNAs annotated on each branch

**Fig. 3:**
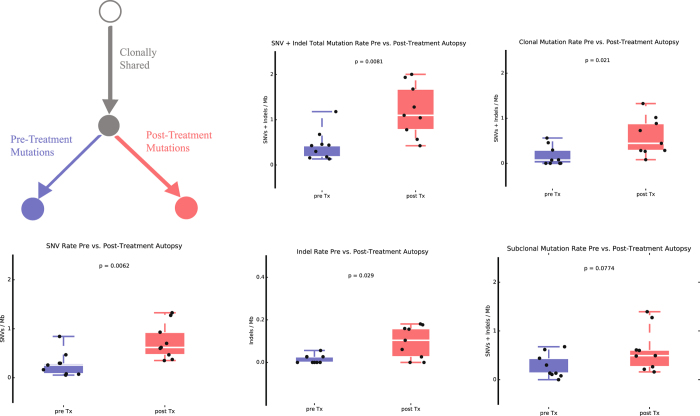
SNV and indel frequencies per sample (/Mb) in cases with pre-treatment primary and post-treatment autopsy sample (*n* = 10)

Even though no tumor met the formal criteria for hypermutator phenotype^23^, there was significant genetic divergence in the post-treatment autopsy samples (Fig. 2a-c, Supplementary Figure 1A-I), with higher post-treatment specific mutation rates (single nucleotide variants, or SNVs, per Mb and small insertions and deletions, or indels, per Mb) compared to the mutation rates respectively in pre-treatment samples (mean 1.202 vs. 0.439; *p* = 0.0081, Mann-Whitney test) (Fig. 3, Supplementary Table 1). We could not detect distinct mutational signatures in mutations detected only in the post-treatment autopsy samples, although we did detect significantly more indels in post-treatment autopsy samples than in pre-treatment samples (mean 0.096 vs. 0.015; *p* = 0.029, Mann-Whitney test), in addition to the overall higher mutation rate (Fig. 3). Since all patients received radiation as part of their care, we speculate that these are radiation-driven indels. Recognizing the possibility that treatment with temozolomide may in part account for the mutation rates observed in the post-treatment autopsy samples, we examined the overall mutational signature in these samples. The characteristic CpC > T temozolomide signature was not detected in any case, suggesting that factors other than exposure to temozolomide contributed to the observed genetic divergence. Detailed evaluation of the mutational signatures specific to pre-treatment and post-treatment autopsy samples uncovered that cytosine to thymidine transitions were predominant in both pre-treatment and post-treatment autopsy cases, which can be attributed to the mutational signature associated with spontaneous deamination of methylated cytosines which occurs naturally and is associated with number of cell divisions^28^, the so-called Signature 1 or “aging” signature (Supplementary Figure 2).

The phylogenetic reconstruction of GBM provided potential insights into possible mechanisms of resistance to different therapies. Below we describe three representative cases that represent a range of treatment regimens. In one case (Fig. 2a: GS-15), a patient diagnosed with an *IDH* wildtype, *MGMT* promoter methylated, right frontal GBM, underwent a gross total resection, treatment with concurrent temozolomide/radiation, and 12 months of adjuvant temozolomide. 6 months after completion of adjuvant treatment, the patient progressed, was treated with dacomitinib (an oral irreversible tyrosine kinase inhibitor of human epidermal growth factor receptors, including EGFR and ERBB2), experienced clinical and radiographic progression after 2 months of therapy, and ultimately died. The pre-treatment and post-treatment autopsy sample shared a common ancestor with shared deletions (del9p21.3, del10), amplifications (focal *EGFR* amplification, amp7, amp19) and mutations (ERBB2 E744K, *TERT* promoter). The pre-treatment sample harbored an additional broad chr20 amplification and the post-treatment autopsy sample had additional alterations, including a clonal six base pair in-frame deletion in *EGFR* (p.C558_P560delinsS) which has not been previously described. The *EGFR* indel may represent a resistance mechanism that arose during treatment with the EGFR inhibitor. Evolutionarily, this case illustrates a late phylogenetic event (*EGFR* indel) altering the dominant treatment-resistant clone, forming a “sequential” narrative of recurrence that is commonly envisioned as the source of treatment resistance.

A second patient (Fig. 2b: GS-02) underwent a resection of a frontal GBM, followed by combined radiation and temozolomide then temozolomide for 6 months and after a clinical and radiographic response, remained stable off of treatment for 6 months, when he was found to have recurrent disease. He was initiated on a clinical trial of a MET inhibitor after his tumor was found to have a c-MET amplification. He progressed after 2 weeks of therapy, and was initiated on bevacizumab and temozolomide with continued progression, and he ultimately died 1 month later. The primary and post-treatment autopsy tumor shared a *TERT* promoter mutation, copy-loss of chromosome 10, and copy-gain of chromosome 7. Thereafter, phylogenetic parsimony indicates that the pre-treatment and post-treatment samples have a branched “sibling” relationship; neither branch is a subclonal descendent of the other. Notably, the primary tumor had a clonal high-level amplification of *MET* (with an average of over 70 copies of MET per cancer cell) that was not present in the post-treatment autopsy sample after treatment with the MET inhibitor. Thus, these data suggest that the primary tumor was in-effect spatially heterogeneous, containing a minor reservoir of *TERT* mutant, chr10 lost, chr7 gain, *MET* non-amplified precursor cells that escape targeted therapy.

Similarly, a third patient (Fig. 2c: GS-06) underwent a subtotal resection of a right temporal lobe GBM, followed by adjuvant radiation, temozolomide and an mTOR inhibitor and had progressive disease 7 months later. He was initiated on the VEGF inhibitor tivozanib for 2 months with poor tolerance, and subsequently transitioned to a different VEGF inhibitor (bevacizumab). He progressed after 4 months of bevacizumab monotherapy and died. His pre-treatment and post-treatment autopsy tumors had shared deletions in 9p21.3, 22q, and chromosome 10, amplification of chromosome 7, and a mutation in the *TERT* promoter. The pre-treatment sample had a high-level focal *EGFR* amplification, whereas the post-treatment autopsy sample did not have the EGFR amplification and had a number of additional mutations and copy-number alterations including a high-level focal *MET* amplification. Increased c-Met expression and activity may play a role in resistance during antiangiogenic therapy^29–31^. It is possible that after receiving targeted therapy, episomal EGFR amplifications may have been selected against as a result of the treatment^32^. However, the presence of 17/152 total mutations specific to the primary (vs. 70/152 mutations in the truncal MRCA clone, and 65/152 total mutations specific to the relapse) suggests evolutionarily that the recurrence is not merely a sequential loss of episomal DNA, but rather outgrowth of a related subclone. These latter representative examples (Figs. 2b,c) highlight the late emergence of a highly-divergent clone derived from a shared precursor pre-existing within a treatment-resistant reservoir.

## Discussion

We analyzed the genetic landscape of GBM at the extremes of disease course using our unique biospecimen resource of autopsy specimens. Comparing pre-treatment and autopsy specimens, we demonstrated a common core of four early genetic events (loss of chr10, chr9p21, gain of chr7 and *TERT* promoter mutations), occurring before the divergence of primary tumor and post-treatment tumor, which are detectable in virtually every case.

More generally, our results suggest that there is significant inter-patient heterogeneity with respect to protein coding (exonic) mutations and that the key early events in GBM phylogeny are not mutations in exonic regions. Exonic mutations in genes such as *TP53* and *EGFR*, although well-established and thought of as key clinically-actionable mutations leading to recurrence and progression, were found in our cohort primarily as later events with respect to GBM phylogeny, as evidenced by their presence on both branches of the evolutionary tree and absence on its trunk. A number of oncogenic alterations emerged after treatment with targeted therapies, indicative of ongoing genomic evolution.

In contrast, we show that *TERT* promoter mutations are present in nearly all cases, from pre-treatment and at the time of death. Interestingly, in a single case the *TERT* promoter mutation was initially subclonal and later became clonal, whereas the copy gains and losses were clonal throughout, suggesting that the *TERT* promoter mutation did not precede the copy number alterations, and was therefore not a requirement for copy gains and losses. Indeed, unlike other brain tumors such as meningomas^33^ where copy number variations arise late during disease progression, our data suggest that copy number changes are amongst the earliest drivers of gliomagenesis.

Collectively, these core four alterations define the shared-origin cell population from which later-emerging recurrences arise in the majority of patients with GBM. Further studies may identify additional truncal alterations contained within the shared-cell precursor. Nevertheless, these alterations point towards the significance of non-coding and structural alterations in gliomagenesis, with significant implications for treatment strategy. Therapies that target protein coding mutations have efficacy in other cancers but lack durable activity in GBM.^34–36^ Our findings indicate this may be because these therapies do not target the entire cellular reservoir, primarily characterized by non-coding and structural changes. Comprehensive analysis of the functional roles of these early events and development of novel therapeutic strategies to target them should be given priority.

## Methods

We identified 12 GBM cases with temporally (*n* = 10) and spatially (*n* = 2) distinct tumor tissue. The study was conducted in accordance with the Declaration of Helsinki. The study was reviewed and approved by the human subjects institutional review board of the Dana-Farber Cancer/Harvard Cancer Center. All patients provided written informed consent for genetic analysis. A board-certified neuropathologist (M.F.) confirmed the histologic diagnoses and selected representative formalin fixed paraffin embedded samples that had an estimated purity of greater than or equal to 40%.

### Sequence data generation and pre-processing

Whole exome sequencing was performed using the sequencing platforms at the Broad Institute. Details of whole exome library construction have been previously described.^37^

A binary SAM file (BAM) file was generated for each sample using the sequencing data processing pipeline known as “Picard” (http://broadinstitute.github.io/picard/). Picard consists of four previously described^38^ steps, detailed below.

#### (1) Alignment to the genome

Alignment was performed using BWA^39^ (http://bio-bwa.sourceforge.net/) to the NCBI Human Reference Genome GRCh37/hg19. The reads in the BAM file are sorted according to their chromosomal position. Unaligned reads are also stored in the BAM file such that all reads that passed the Illumina quality filter (PF reads) are kept in the BAM.

#### (2) Base-quality recalibration

Each base is associated with a Phred-like quality Q score representing the probability that the base call is erroneous. The Q score represent −10*log (probability of error), rounded to an integer value. In order to make sure that Q30 bases indeed have a 1 in a 1000 chance of being wrong we used a GATK tool (http://www.broadinstitute.org/gatk) that empirically recalibrates the qualities based on the original Q score (generated by the Illumina software), the read-cycle, the lane, the tile, the base in question and the preceding base. The original quality scores are also kept in the BAM file in the read-level OQ tag.

#### (3) Aggregation of lane and library-level data

Multiple lanes and libraries are aggregated into a single BAM per sample. Lane-level BAM files are combined to library-level BAM files that are then combined to sample-level BAM files. The BAM files contain read-groups that represent the library and lane information. Information regarding the read groups appears in the BAM header (see the BAM file specifications in http://samtools.sourceforge.net/SAM1.pdf).

#### (4) Marking of duplicated reads

Molecular duplicates are flagged using the MarkDuplicates algorithm from Picard (http://broadinstitute.github.io/picard/). The method identifies pairs of reads in which both ends map to the exact same genomic position as being multiple reads of the same DNA molecule and hence marks all but the first as duplicates.

#### Targeted sequencing of *TERT* promoter region

Targeted sequencing of the *TERT* promoter region was also performed for each tumor sample using Fluidigm sequencing technologies. A portion of the *TERT* promoter region [273 bp; Chr5: 1,295,040–1,295,313 (hg19)] was amplified and sequenced in 20 samples. These PCRs were carried out in two reactions. Round-1 PCR primers contained target-specific sequences and Illumina adapter sequences, producing a product of 341 bp. Round-2 PCR was a “tailing” PCR in that PCR2 primers contained overlap of the Illumina adapter sequence, as well as flow cell attachment sequence, and an eight bp index on the reverse primer between the adapter sequence and flow cell attachment sequence. This tailing PCR produced sequence-ready constructs of 398 bp that did not require further library construction. First-round PCR was carried out using the Platinum Pfx DNA polymerase kit (Life Technologies, Inc.). PCR1 reactions consisted of 50 ul: 2 ul DNA (at ~25 ng/ul), 3 ul mixed F/R tailed target-specific primer (at 20 uM mixed), 5 ul 10X Pfx amplification buffer, 1.5 ul dNTPs [at 10 mM each (Agilent Technologies)], 0.8 ul Pfx Platinum DNA polymerase, 1 ul MgSO_4_ (at 50 mM), 5 ul 10X Pfx Enhancer Solution, and 31.7 ul nuclease free water. The polymerase (0.4 ul polymerase + 1.6 ul water) was added to reactions after 1 min at 95 °C. Thermal cycling consisted of 95 °C for 5 min (paused at 1 min to add polymerase), 30 cycles of [95 °C 30 sec, 55 °C 30 sec, 68 °C 1 min]. A sampling of PCR1 products (and negative control) were visually inspected on the Lab Chip GX II Caliper Instrument (Perkin Elmer). Next, second-round index-tailing PCRs were carried out using the HiFi Library Amplification kit (Kapa Biosystems, Inc.). PCR2 reactions consisted of 60 ul: 10 ul PCR1 product, 12 ul 5X Kapa HiFi Fidelity Buffer, 1 ul dNTPs (25 uM), 1 ul Kapa HiFi HotStart Enzyme, 32 ul nuclease free water, and 4 ul PCR2 F/R index-primer mix (25 uM mixed, plate of 96). Thermal cycling consisted of 98 °C for 45 sec, 8 cycles of [98 °C 15 sec, 60 °C 30 sec, 72 °C 30 sec] and 1 min at 72 °C. Indexed amplicons were pooled in equal volumes (96 reactions per pool), and purified using 1.5X solid-phase reversible immobilization (SPRI) cleanup with Agencourt Ampure XP beads (Beckman Coulter). Final amplicon library pools were visually inspected and quantified on a BioAnalyzer (Agilent Technologies). The library was re-quantified by SYBR green qPCR before denaturing and cluster generation. PhiX library, derived from the well-characterized and small PhiX genome, was spiked in at 15% to add diversity to single-amplicon clusters for improved cluster imaging. One MiSeq run (2 × 150 bp paired end with standard sequencing primers) was carried out for each pool of indexed amplicons, using standard sequencing protocols (Illumina).

### Cancer genome analysis pipeline

Whole exome sequencing data was analyzed using Firehose (developed at the Broad Institute; https://www.broadinstitute.org/cancer/cga). All tumor-normal pairs passed the Firehose QC pipeline, which included testing for DNA contamination of a sample from other individuals using the Contest algorithm,^40^ as well as cross-checking lane fingerprints. A more detailed description of the QC pipeline can be found here.^38^

#### Identification of somatic single nucleotide variants (SSNVs) and small insertions and deletions (indels)

Candidate SSNVs were detected using the point mutation calling algorithm MuTect,^41^ ran on each tumor-normal pair. All mutations were filtered using the oxoG filter, which filters mutations that arise due to oxidation of a G base pair on only one strand during fragmentation.^42^ Since the tumor samples analyzed for this study were formalin-fixed paraffin-embedded (FFPE) samples, candidate SSNVs were then filtered using a Panel of Normals (PoN) filter, comprised of 374 FFPE normal samples. This step was taken to remove potential sequencing artifacts and potential germline sites missed in the matched normal sample. Mutant and reference allele counts were also estimated at known hotspot mutation sites in the TERT promoter region (p.C228T and p.C250T) in the Fluidigm targeted sequencing BAM files. Candidate indels were detected using the Strelka indel calling algorithm^43^ on each tumor-normal pair. Similarly to SSNV filtering, indel calls were filtered using the same PoN filter. While many artifactual mutations were removed in the various filtering processes, we still manually reviewed all validated mutations to remove further artifacts, which included mutations called on low mapping quality reads, mutations called on reads which also contained indels and other low allelic fraction point mutations, mutation supported only by duplicate reads, mutations with strong orientation bias, as well as mutations called in poorly mapping regions. In total, 141 mutations (3 indels, 138 SSNVs) were manually filtered across 1,476 mutations calls from all 12 patients (filter rate of 9.3%).

#### Identification of somatic copy number alterations (SCNAs)

A coverage profile for each tumor sample was estimated using the ReCapSeg tool (http://gatkforums.broadinstitute.org/gatk/discussion/5640/recapseg-overview). This tool works by first normalizing read coverage over each target segment with the total number of aligned reads. Next, coverage at every segment is normalized against the coverage across a Panel of Normals (PoN) generated from 25 normal FFPE samples sequenced using the same target regions. Next, target regions are merged to form segments corresponding to the same copy number event using the circular binary segmentation algorithm.^44^ Allelic copy ratio was then estimated by measuring allelic fraction of germline heterozygous SNPs in each tumor sample (found in matched normal samples), and combining these estimates with the observed copy ratio of each segment using the AllelicCapseg tool (http://archive.broadinstitute.org/cancer/cga/acsbeta). Finally, somatic copy number alterations were estimated by running the ABSOLUTE algorithm,^12^ which maps allelic copy ratios to allelic copy numbers via a linear transformation after correcting for purity and ploidy of the sample.

#### Calculation of cancer cell fractions (CCFs) of SSNVs and indels, and subsequent phylogenetic analysis

The CCF distribution of each point mutation (both SSNVs and indels) was estimated using ABSOLUTE. Point mutations were force-called across each tumor sample belonging to each patient; a process in which the aggregate set of all point mutations found in each tumor sample belonging to a patient was formed, and the mutant and reference allele counts in each sample estimated using samtools (http://samtools.sourceforge.net/). Reads were only included if they had a unique pair, had mapping quality greater than or equal to 5, and a base quality at the site of interest greater than or equal to 20. The mutant and reference allele counts for each mutation in the force-called set of mutations was used as input to ABSOLUTE, which estimates the CCF distribution of each point mutation based on purity and local ploidy of the site.

Mutation CCFs were subsequently clustered across each individual using a Bayesian clustering method. The final clusters were found by sampling from a Dirichlet process using a Markov chain Monte Carlo (MCMC) sampler, as described here.^24, 45^ Five hundred MCMC iterations were used to find the final number of clusters. Phylogenetic trees were then drawn for each patient based on the CCF estimates of these clusters.

While our resolution to detect mutations at low cancer cell fraction is limited, we can still estimate our power to detect mutations at a CCF of 0.05 given the tumor’s purity and local absolute copy number, calculated as follows. For a mutation with coverage C (total number of reads mapping to locus of mutation) in a sample with purity P, and total number of allelic copies in the region containing the mutation, N, we calculate the expected allele fraction of mutation with CCF of 0.05 as:

$$af{\rm{ = }}\frac{{0.05{\rm{*}}P}}{{2{\rm{*}}\left( {1 - P} \right) + N{\rm{*}}P}}$$

The power to detect a mutation at CCF of 0.05 at that locus is then:

$$\mathop {\sum }\limits_{i = 1}^C Pr\left( {i;\,C,\,af} \right),\,where\,Pr\left( {k;\,n,\,p} \right)\,is\,the\,binomial\,p.m.f{\rm{.}}$$

We found that we had greater than 50% power to detect mutations at CCF of 0.05 in 5 point mutations in driver genes which were found in a clone not present in the primary but clonal in the metastasis (MSH6_p.F11Y in PKB-GS-001, PTEN_p.I33T in PKB-GS-005, EGFR_p.A289V in PKB-GS-009, SPEN_p.P3434S in PKB-GS-015, and EGFR_p.558_ in PKB-GS-015), while 2 point mutations in driver genes had power to detect less than 50% in similar clones (EGFR_p.C636F in PKB-GS-005 and MICALCL_p.G245fs in PKB-GS-005). Although the MSH6_p.F11Y had one supporting read in the primary sample in PKB-GS-001, it clustered with a clone with estimated CCF of 0.01, below our threshold of subclonal presence of a clone.

### Data availability statement

Supplementary Table 2 includes the mutation annotation format (MAF) file for all patients sequenced. Sequence data that support the findings of this study have been deposited in dbGaP with the accession code phs1424.v1.p1.

## Electronic supplementary material

Supplementary Figures and Table

Supplementary Table 2

## Electronic supplementary material

**Supplementary Information** accompanies the paper on the *npj Precision Oncology* website (doi:10.1038/s41698-017-0035-9).
