# Supplementary material for: Resolving the phylogenetic origin of glioblastoma via multifocal genomic analysis of pre-treatment and treatment-resistant autopsy specimens
Source: NPJ Precis Oncol. 2017 Sep 18;1:33. doi: 10.1038/s41698-017-0035-9 (PMC5871833; doi:10.1038/s41698-017-0035-9)
Supplement: Supplementary file 1 — Supplementary Figures and Table [file 41698_2017_35_MOESM1_ESM.pdf]

Supplementary figures

Supplementary Figure 1A

GS-10

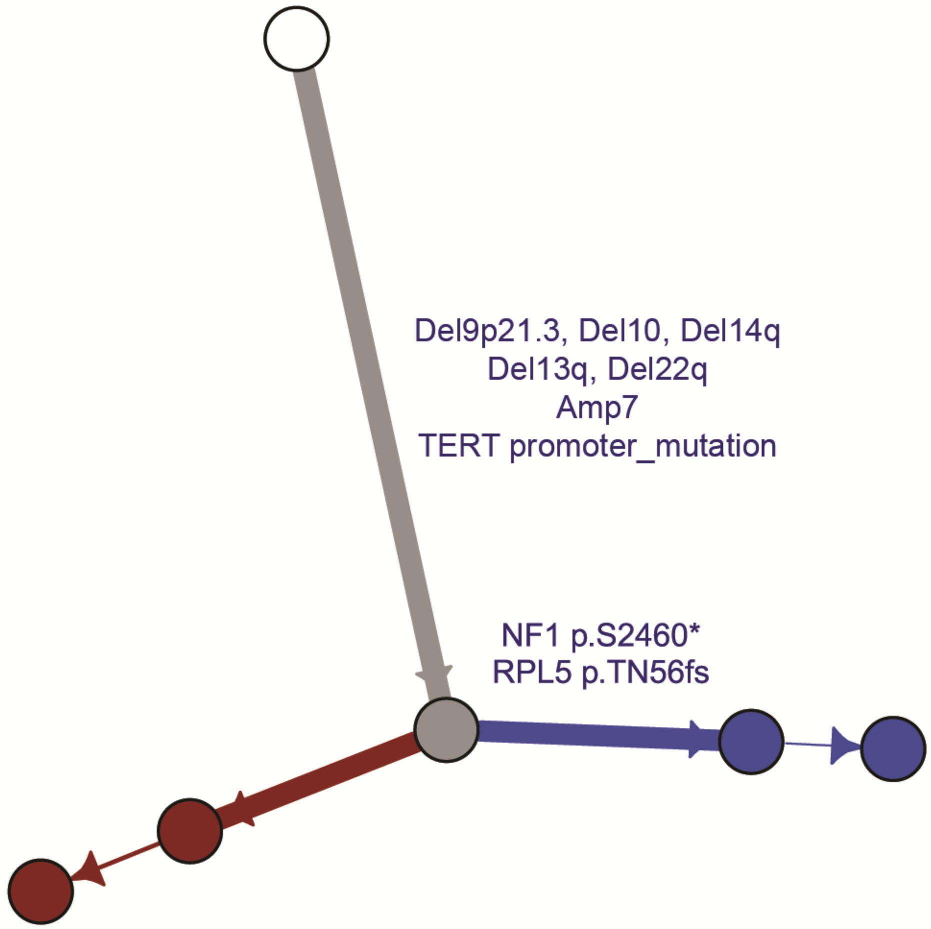

Supplementary Figure 1B

GS-03

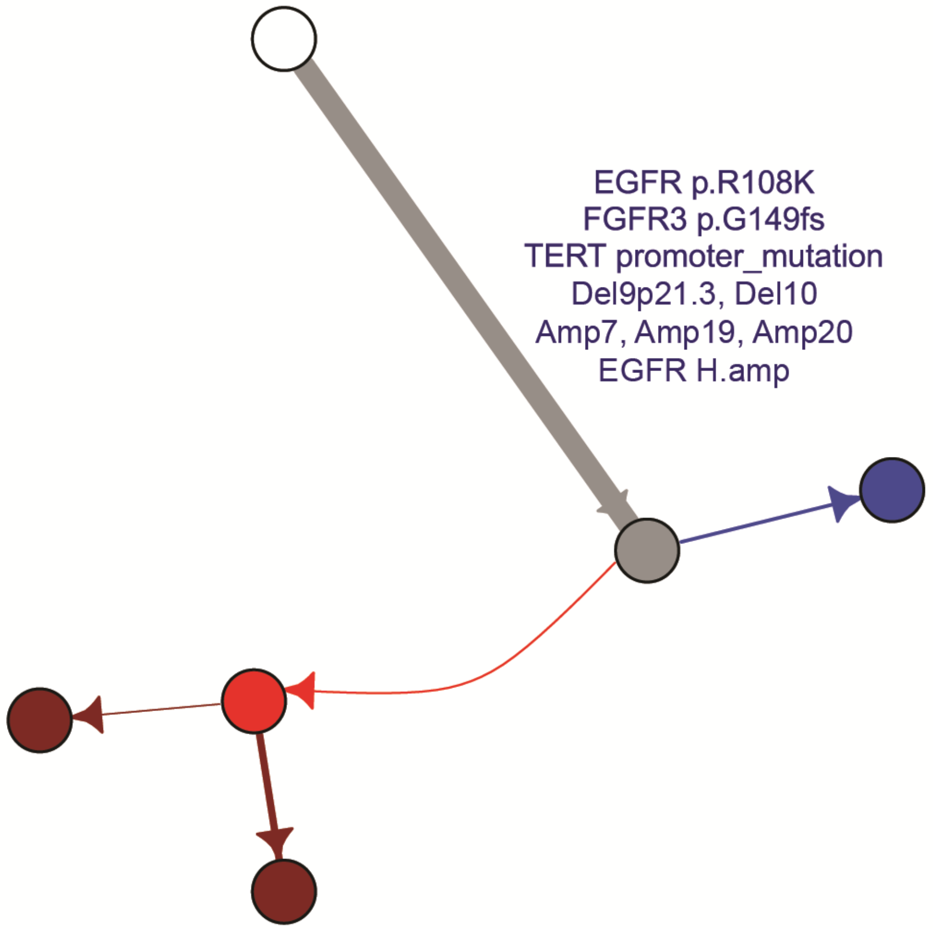

Supplementary Figure 1C
GS-05

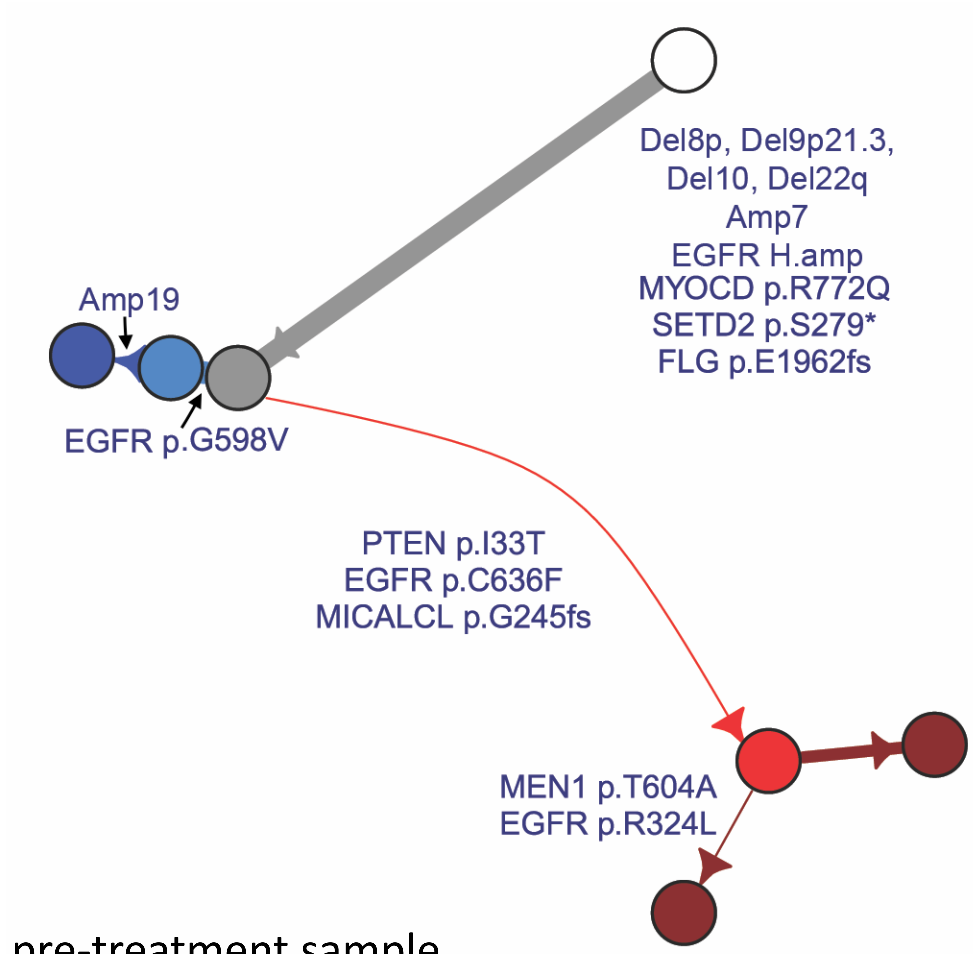

No fluidigm data for the pre-treatment sample
TERT clonal in post-treatment sample

Supplementary Figure 1D
GS-07

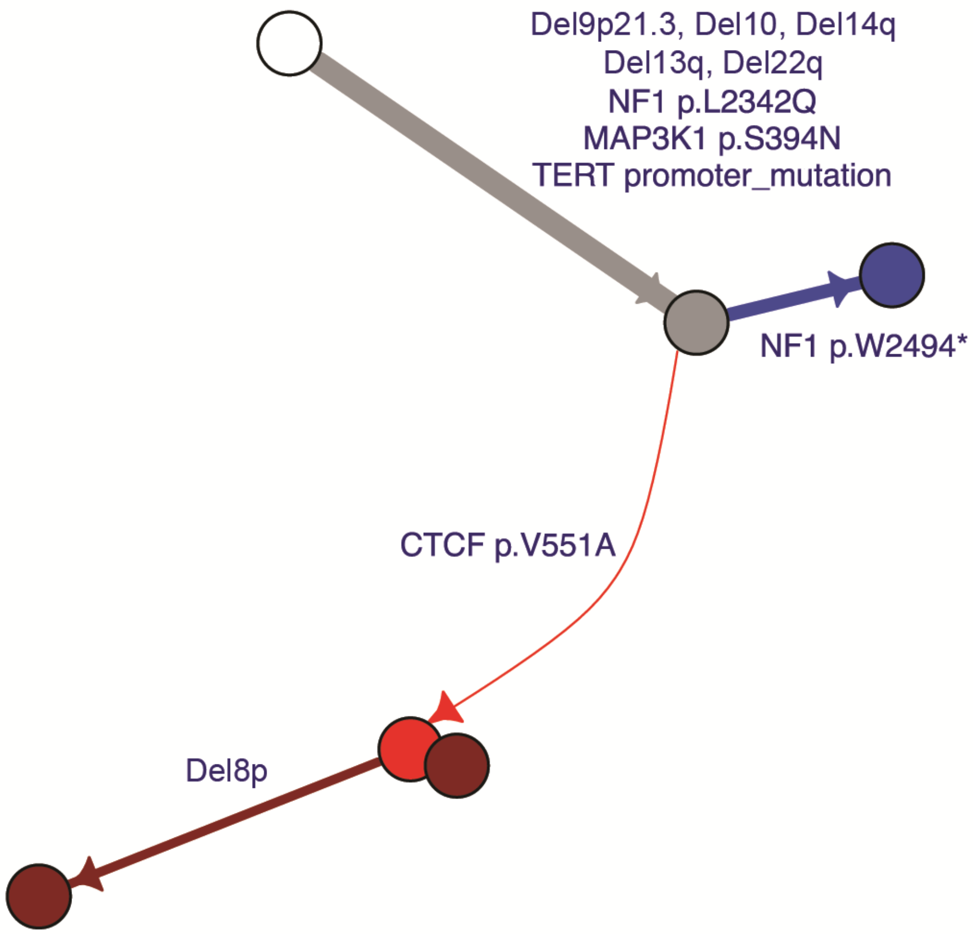

Supplementary Figure 1E
GS-01

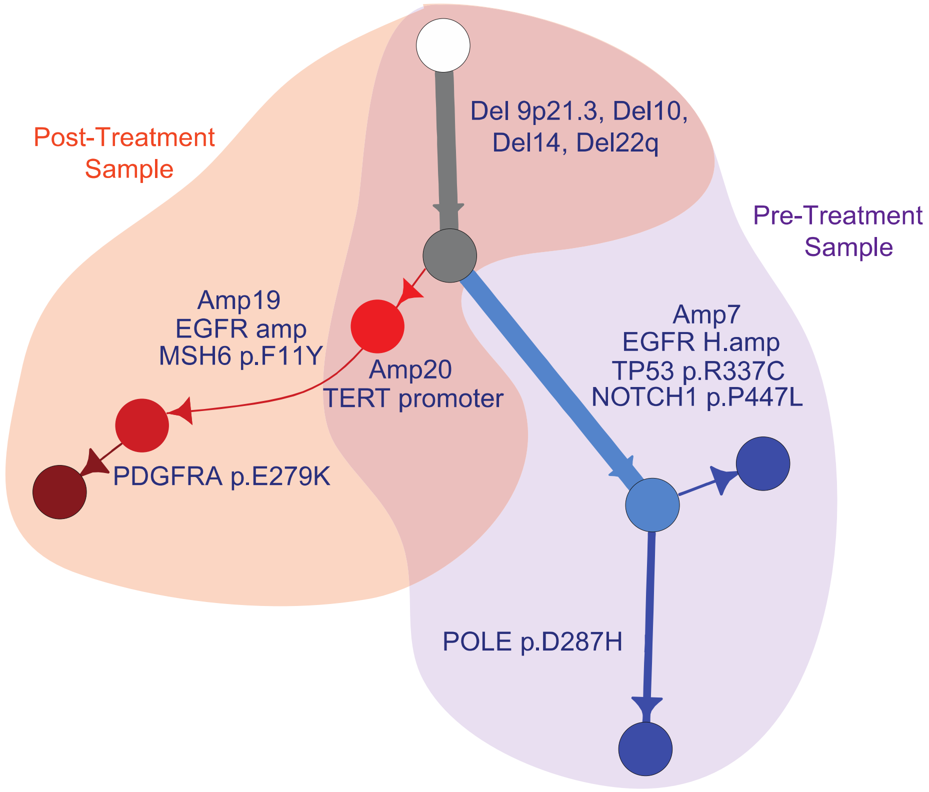

Supplementary Figure 1F
GS-14 (LUNG)

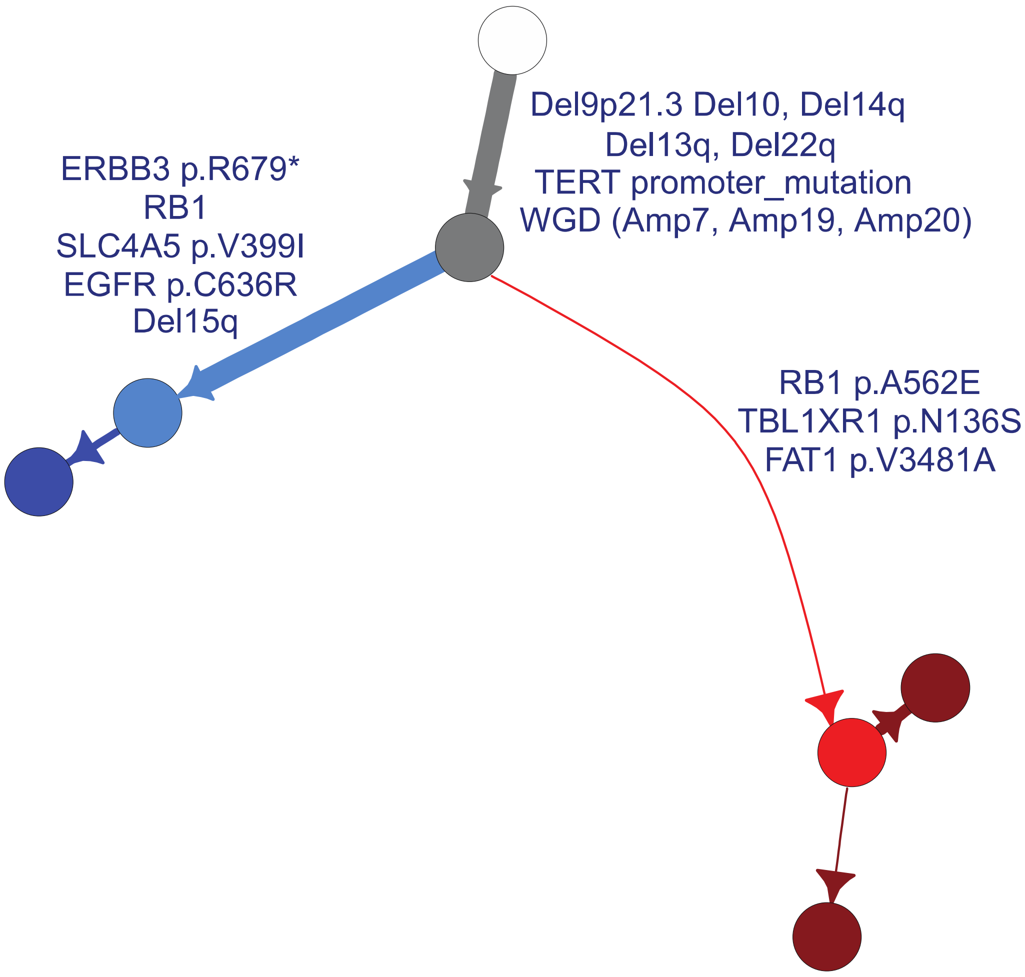

Supplementary Figure 1G
GS-08 (LUNG)

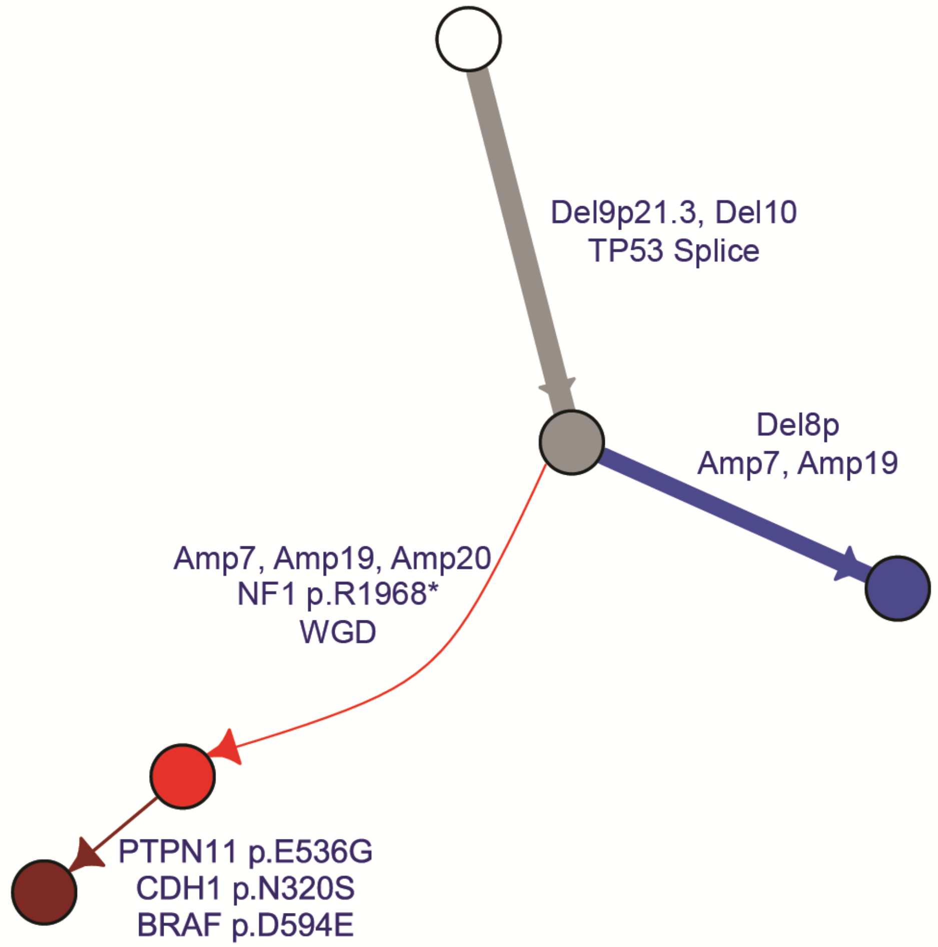

Supplementary Figure 1H
GS-09

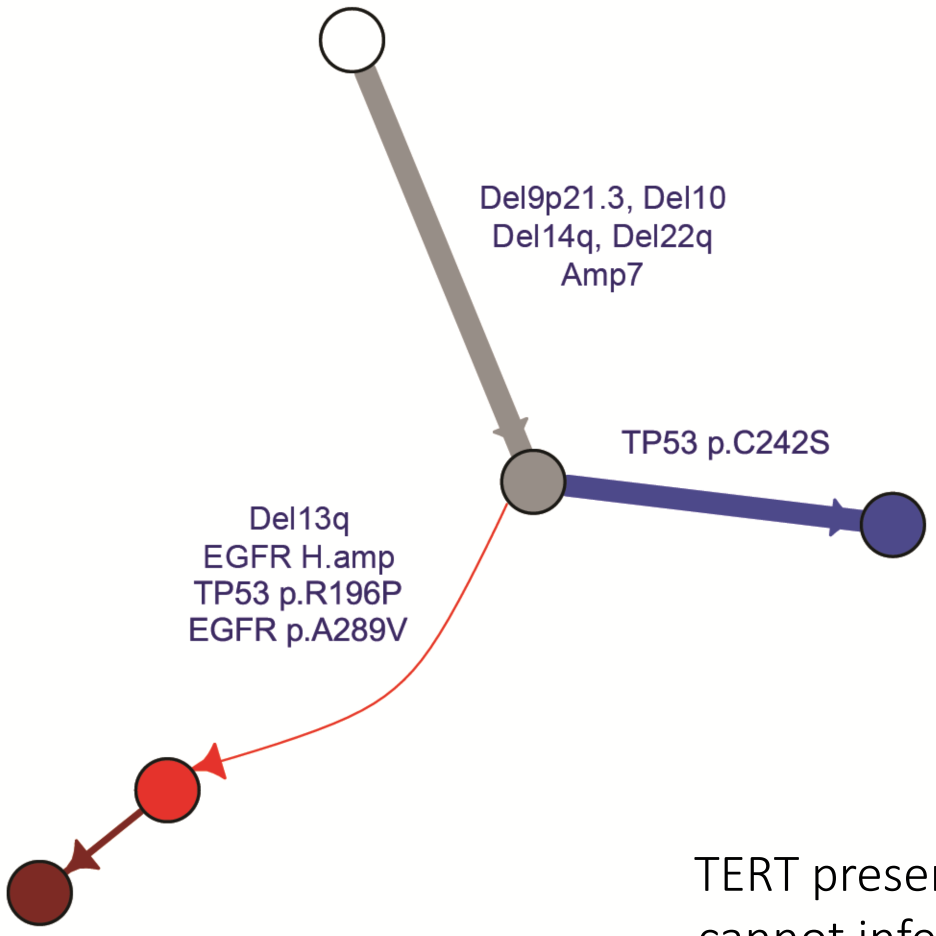

TERT present in both samples, cannot infer clonality because different DNA aliquots

Supplementary Figure 1I
GS-11

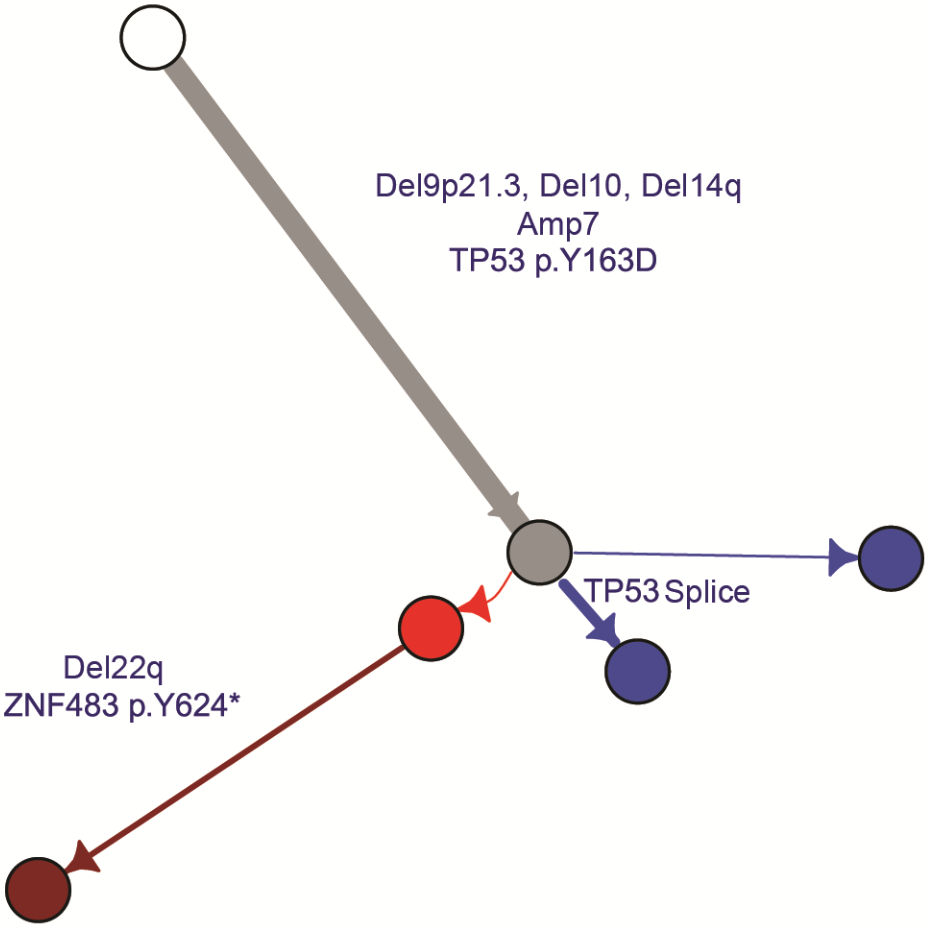

Supplementary Figure 2

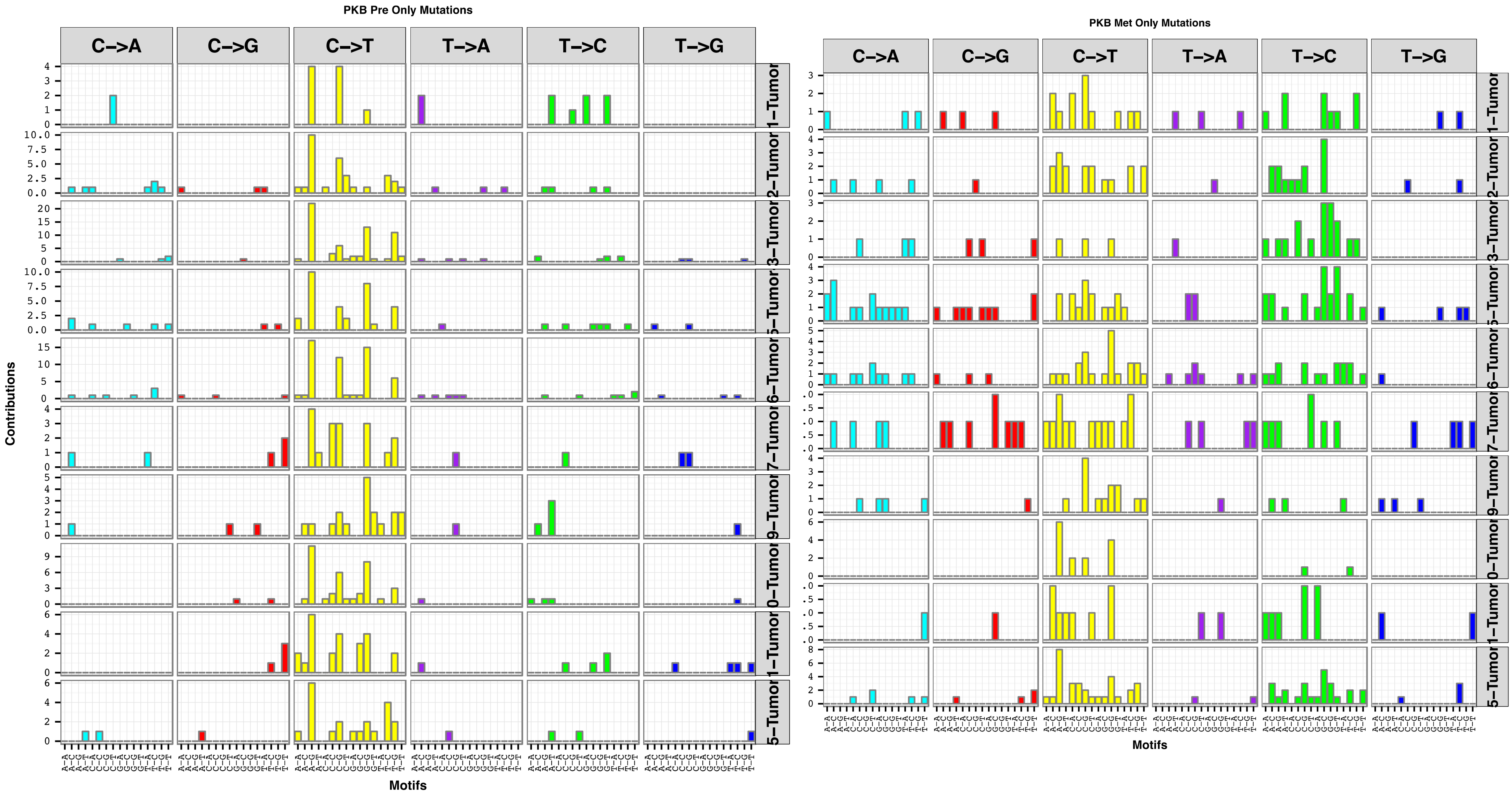

Supplementary Table 1: Mutation Rates

Mutation Rate Comparison	Pre-treatment			Post-treatment autopsy			p-value (Mann-Whitney)
	Mean	Minimum	Maximum	Mean	Minimum	Maximum	
All mutations	0.4391	0.1336	1.1796	1.2015	0.4255	2.0028	0.0081
Clonal mutations	0.1627	0.0000	0.5617	0.5907	0.0809	1.3267	0.021
Subclonal mutations	0.2764	0.0000	0.6762	0.6108	0.1596	1.3947	0.0774
SNVs	0.2808	0.0534	0.8425	0.7379	0.3508	1.3265	0.0062
Indels	0.0151	0.0000	0.0562	0.0960	0.0000	0.1808	0.029
